# Protein Dynamics in Plant Immunity: Insights into Plant–Pest Interactions

**DOI:** 10.3390/ijms252312951

**Published:** 2024-12-02

**Authors:** Yan Zhao, Yanru Wang

**Affiliations:** 1 State Key Laboratory of Hybrid Rice, Hunan Hybrid Rice Research Center, Hunan Academy of Agricultural Sciences, Changsha 410125, China; 2 Department of Plant Genetics and Breeding, China Agricultural University, Beijing 100193, China

**Keywords:** plant resistance, pest, protein regulation, interaction

## Abstract

All living organisms regulate biological activities by proteins. When plants encounter pest invasions, the delicate balance between protein synthesis and degradation becomes even more pivotal for mounting an effective defense response. In this review, we summarize the mechanisms by which plants regulate their proteins to effectively coordinate immune responses during plant–pest interactions. Additionally, we discuss the main pathway proteins through which pest effectors manipulate host protein homeostasis in plants to facilitate their infestation. Understanding these processes at the molecular level not only deepens our knowledge of plant immunity but also holds the potential to inform strategies for developing pest-resistant crops, contributing to sustainable and resilient agriculture.

## 1. Introduction

Plants are attacked by parasitic pests, including insects and nematodes, causing considerable economic losses worldwide. Instead of sitting still, plants have developed a multilayered immune system to defend against invasion of pathogens and pests [1]. This includes immune receptors to detect specific molecular patterns and effectors. These receptors, located on the plant cell surface or within the cell, act as sentinels, constantly surveilling the environment for signs of danger [1,2]. Upon recognition of pathogen/pest-derived molecules, these receptors trigger a rapid and intricate signaling cascade, activating various downstream defense responses, including calcium ion flux, production of reactive oxygen species, activation of mitogen-activated protein kinase (MAPK) cascades, and hormone signaling and transcriptional reprogramming [2,3]. These processes require a high degree of proteomic plasticity involving both the synthesis and turnover of proteins.

Protein homeostasis, also known as proteostasis, refers to the dynamic equilibrium maintained within a cell or organism concerning the synthesis, folding, assembly, trafficking, and degradation of proteins [4]. Protein homeostasis can be influenced by factors such as protein translation, folding, localization, post-translational modifications, proteasomes, and autophagic activity [5]. Maintaining protein homeostasis is crucial for various cellular processes, as proteins play essential roles in structural support, enzymatic activity, signaling pathways, and many other biological functions. Upon detecting the presence of pests, the cellular machinery orchestrates the synthesis of defense-related proteins, such as pathogenesis-related proteins and protease inhibitors, which play crucial roles in deterring and combating herbivorous invaders [6,7]. These proteins not only serve as direct deterrents or toxins against pests but also contribute to signaling cascades that amplify the overall defense response. Simultaneously, the plant’s protein degradation pathways, including the ubiquitin–proteasome system (UPS) and autophagy, play a vital role in removing damaged or misfolded proteins that may accumulate as a result of stress induced by pest attacks [8,9,10,11]. This ensures that the cellular environment remains conducive to efficient defense responses, facilitating the rapid deployment of defense molecules.

In the ongoing evolutionary arms race between plants and pests, the latter have developed sophisticated strategies to counteract plant’s intricate defense mechanisms. One notable tactic employed by pests involves the secretion of specific proteins known as effectors [12]. These effector proteins serve as molecular tools that pests utilize to subvert and manipulate various components of plant immune system [12,13]. By doing so, pests can suppress or hijack immune responses, allowing them to bypass plant defenses. Plants have evolved “guard” proteins that detect pathogen effectors by monitoring the host targets they manipulate [14]. However, to counteract this, some pathogens developed decoy effectors that closely resemble the host targets or actual effector proteins. These decoys are recognized by the plant’s guard proteins, diverting the immune response away from the genuine effectors [14,15]. This misdirection helps pathogens avoid triggering strong immune reactions and highlights a co-evolutionary dynamic where both plant and pathogen continuously adapt their strategies. Currently, there are no direct reports of “decoy” effectors in plant–pest interactions; however, the presence of multiple effector gene copies, some of which are non-enzymatic or mimic host targets, suggests a potential decoy strategy in plant–pest interactions [16]. This adds an additional layer of regulation to the plant’s protein networks, impacting broader physiological processes.

In this review, we focus on the regulation of plant proteins during plant–pest interactions (specifically insects and nematodes), with an emphasis on the dynamics at the protein level. We address two key areas: firstly, we summarize the mechanisms by which plants regulate protein production and degradation, highlighting the complex pathways and signaling cascades involved in maintaining cellular homeostasis and mounting effective immune responses. Concurrently, we focus on the primary strategies employed by pest effectors to disrupt and manipulate plant protein homeostasis, providing insights into how pests subvert these regulatory systems to their advantage.

## 2. Plant Protein Regulations in Response to Pest Invasion

**Figure 1:**
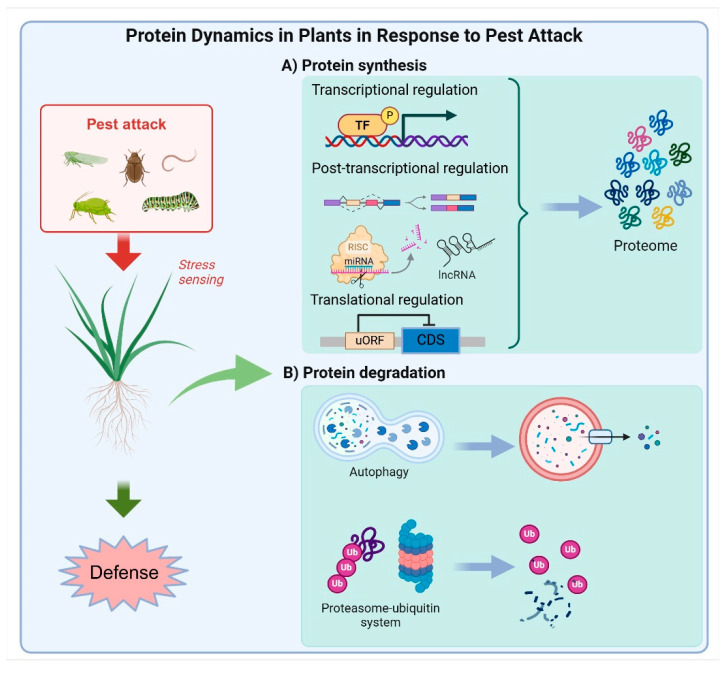
Protein dynamics in plants in response to pest attack. Upon pest invasion, transcriptional, post-transcriptional, and translational controls regulate the synthesis of immune-related proteins, ensuring their timely production during pest-induced stress (**A**). Concurrently, protein degradation pathways, including the ubiquitin–proteasome system (UPS) and autophagy, remove damaged or excess proteins, shaping plant defense mechanisms against pests (**B**).

Protein abundance within the cell is a critical determinant of the efficacy and duration of signaling responses during plant–pest interactions. The precise regulation of key proteins, such as receptors and downstream signaling components, is essential for orchestrating effective defense mechanisms against pests. Transcriptional, post-transcriptional, and translational controls are pivotal in governing the synthesis of immune-related proteins, ensuring their timely production in response to pest-induced stresses. Additionally, protein degradation pathways, such as the UPS and autophagy, play crucial roles in eliminating damaged or surplus proteins, further shaping the plant’s defense responses against pests (Figure 1).

### 2.1. Regulation of Protein Production

The regulation of plant protein synthesis in response to insects and nematodes differs due to their distinct feeding strategies and modes of interaction with the host. Chewing herbivores, such as cotton bollworms and diamondback moths, inflict extensive tissue damage by consuming plant leaves, which rapidly activates phytohormone signaling, especially jasmonic acid (JA) signaling [17]. This response leads to the production of defense proteins like proteinase inhibitors and various secondary metabolites that deter herbivory [17,18]. Piercing-sucking insects, such as aphids and planthoppers, insert their stylets into plant tissues to feed on nutrient-rich phloem sap. This feeding strategy, often affecting both the JA and salicylic acid (SA) pathways, not only depletes essential nutrients but also disrupts the plant’s physiological processes, often resulting in stunted growth and reduced vitality of the host plant [19]. These insect pests typically inflict damage over a relatively short period as they feed and move to new sites. In contrast, plant–parasitic nematodes, such as cyst and root-knot nematodes, establish long-term feeding sites by secreting effectors that reprogram host protein synthesis, promoting the formation of specialized structures (e.g., syncytia or giant cells). Nematodes manipulate hormonal signaling (auxins, cytokinins) to enhance the synthesis of cell wall-modifying enzymes, nutrient transporters, and growth-related proteins [20,21]. While insect-induced responses focus on immediate defense, nematode-induced changes involve sustained reprogramming of protein synthesis to support nutrient acquisition. Notably, crosstalk between different hormonal pathways, including JA, SA, and auxins, further fine-tunes these defense and adaptation strategies, showcasing the intricate coordination required for effective plant responses.

Synthesis of immune-related proteins is primarily governed by a complex interplay of regulatory processes, prominently including transcriptional regulation, RNA stability, RNA splicing, and translation. These fundamental mechanisms orchestrate the precise expression of genes encoding immune proteins, ensuring their timely production, proper functional diversification, and modulation in response to pests.

Transcriptional regulation is probably the most extensively studied and most commonly recognized target of signal transduction pathways. Numerous studies have reported the transcriptional changes during plant–pest interactions [22,23,24,25,26]. This aspect of regulation can be achieved by modulating the nuclear localization and accumulation of transcription factors (TFs), coactivators, and other regulatory proteins, adjusting the DNA-binding capacities of these proteins, or posttranslationally modifying histones to alter chromatin architecture [27]. Among them, TFs are major players in early and late defense signaling [17]. Various TFs have been reported to be involved in the regulation of plant defense against pests [17,28,29].

The processing and stability of mRNA molecules are also critical determinants of gene expression dynamics, influencing the abundance of proteins involved in plant defense responses against pests. Alternative splicing, in particular, can produce multiple mRNA isoforms with distinct functions, thereby expanding the repertoire of proteins involved in plant defense mechanisms. Several studies have indicated that insect herbivory feeding elicits genome-wide alternative splicing [30,31]. micro-RNAs and other noncoding RNAs, such as long noncoding RNAs, also take part in the control of plant signals against pests [32,33,34,35,36,37]. For example, the ancient and conserved miRNA miR319 has been reported to target different genes and mediate defense against different pests. OsmiR319 targets OsPCF5 to modulate resistance to brown planthopper in rice through association with MYB proteins [36]. The miR319/TCP4 module in tomato affected JA synthetic genes and the endogenous JA level in leaves, thereby mediating root-knot nematode (RKN) resistance [37]. The miR319 positively regulates trichome density in *Populus tomentosa* by targeting TCP19. TCP19 interacts physically with the DELLA protein RGA, forming a regulatory complex that synergistically participates in trichome initiation through gibberellic acid (GA) signaling pathways [35]. Long non-coding RNA also play a role in plant defense against pests. A significant number of long non-coding RNAs (lncRNAs) in tobacco are induced in response to feeding by the phytophagous insect *Manduca sexta*. Among them, silencing the lncRNAs JAL1 and JAL3 was shown to weaken plant resistance to *M. sexta*, probably through their roles in inhibiting the accumulation of JA and JA derivatives.

Translational control of protein synthesis is also an important strategy of immune regulation [38,39,40]. This level of regulation was firstly revealed by the finding that the immune transcriptome was poorly correlated with the translatome induced by the microbe-associated molecular pattern elf18 [39]. Upstream open reading frames (uORFs) are cis-acting elements that primarily inhibit the translation initiation of the downstream primary ORF through ribosome stalling [41]. Combining pathogen-inducible uORF with a pathogen-inducible promoter has been demonstrated to be a powerful strategy for engineering resistance in plants [42,43]. It allows a stringent pathogen-inducible expression of defense proteins without compromising plant fitness. Although the related studies have not been reported in the plant–pest interaction system, future studies focus on exploring the potential application of similar regulatory elements, which will serve as a promising approach for bolstering plant defense against pests.

### 2.2. Protein Degradation

Protein degradation plays a pivotal role in shaping the abundance and dynamics of proteins within cells. This process, often facilitated by proteolytic enzymes and protein degradation machinery, regulates the turnover of proteins, ensuring the removal of damaged, misfolded, or surplus proteins to maintain cellular homeostasis. Post-translational modifications (PTMs) such as phosphorylation, ubiquitination, acetylation, SUMOylation, and glycosylation are involved in the degradation processes, influencing the stability, activity, and localization of immune-related proteins [44,45]. Proteins decorated by different PTMs can be degraded by different machinery at different times. For example, the flagellin receptor Flagellin Sensitive 2 (FLS2) is modified by SUMO, ubiquitin, phosphorylation, N-glycosylation, and S-acylation [4]. It can be degraded by both autophagy and the UPS system [11]. This review mainly focuses on the two principal degradation pathways during plant–pest interaction: the UPS and autophagy. The interplay between autophagy, the UPS system, and PTMs has been reviewed elsewhere [11,45].

#### 2.2.1. The Ubiquitin–Proteasome System

**Figure 2:**
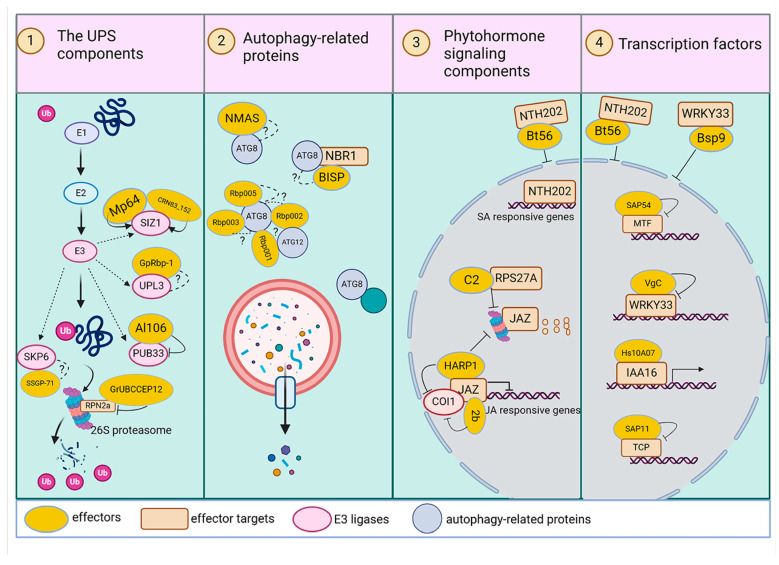
The primary components targeted by effectors from pest and insect-borne microbes to alter the host proteome. Effectors are represented by yellow ovals. They inhibitor /impede (end line) or stabilize (arrow) their targets. Some effectors act on target proteins through mechanisms that remain unknown (question mark). Some effectors target the UPS and autophagy, which are the main protein degradation systems in plants. E3 ligases are the most reported targets in the UPS system. Different E3 ligases are targeted by different effectors. Autophagy-related proteins are also targeted by effectors; the outcomes of these interactions are still to be investigated. Phytohormone signaling is governed by the UPS system and is thus also targeted by effectors from pest and insect-borne microbes. The JA signaling repressor JAZ proteins are the most reported targets. Some effectors hijack transcription factors to modulate gene expression. Effectors such as Bt56 and Bsp9 impede transcription factors moving to the nucleus, thus limiting their function. Transcription factors orchestrate gene expression, and the outcomes of these interactions ultimately determine the composition and dynamics of the proteome within cells.

The UPS is a highly conserved pathway involved in the degradation of up to 80% of eukaryotic proteins [46]. It is particularly important for plants, as reflected by the fact that nearly 6% of the *Arabidopsis* proteome are UPS components [47]. Poly-ubiquitination of target proteins is a prerequisite for recycling proteins through the UPS. Ubiquitination of target proteins is a tightly regulated process controlled by a three-step enzyme cascade involving activation (E1), conjugation (E2), and ligation (E3) [48]. The substrate protein bears one or multiple ubiquitin chains that are then recognized by the 26S proteasome for its subsequent degradation (Figure 2).

UPS regulation of plant immunity affects all stages, from immune receptors to downstream signaling components [49,50]. The ubiquitination-26S proteasome system and the HSP90-SGT1-RAR1 chaperones have been documented as having key functions in the regulation of nucleotide leucin-rich receptor (NLR) stability [44,51]. SGT1 was found to directly interact with SKP1 (S-phase kinase associated protein 1), the core component of the SCF E3 ubiquitin ligase [52]. SGT1 was suggested to act as an adaptor, connecting HSP90 with the SCF complex [53]. Mi-1.2 is an NLR receptor that confers resistance to potato aphids, whiteflies, and root-knot nematodes [13]. By employing virus-induced gene silencing, HSP90 and SGT1 were demonstrated to have a role in Mi-1.2-mediated resistance to aphids [54].

Hormone signaling components are the major downstream immune components targeted by the UPS in plant–pest interaction. To date, every single plant hormone signaling pathway has been shown to be regulated by protein ubiquitination [4]. Hormone signaling plays a pivotal role in plant–pest interaction [55,56]. Thus, regulation of phytohormone signal components has been widely reported in the orchestration of plant resistance to pests [17,57,58,59]. The effectiveness of this system lies in its ability to coordinate internal and external cues with developmental processes through a mechanism that is both rapid and spatially regulated. The mobile nature of plant hormones, combined with the mechanistic precision of the UPS, ensures timely degradation of downstream targets in hormone signaling pathways, enabling plants to respond dynamically to pest challenges while maintaining developmental homeostasis. Jasmonic acid (JA) signaling is especially established as the core pathway [17]. For instance, it was shown that when plants are injured by insect attack, injury rapidly triggers calcium influxes to activate calmodulin-dependent phosphorylation and degradation of the JA negative regulator JAV1 via the UPS. This disrupts the JAV1-JAZ8-WRKY51 (JJW) complex and subsequently activates JA defense responses [57]. The SA pathway master transcriptional regulator non-expresser of PR genes 1 (NPR1) is reported to regulate plant resistance to *Spodoptera litura* [60], whitefly [61], white-backed planthopper [62], and rice striped stem borer [63]. NPR1 stability is also controlled by the UPS [64,65].

Presently, the scope of targets regulated by the UPS in the context of plant–pest interactions remains somewhat limited. Although important regulators of plant defense responses, such as transcription factors (TFs), E3 ubiquitin ligases, and other immune-related proteins, have been identified as targets of the UPS in the context of plant–pathogen interactions, our understanding of UPS-mediated regulation in plant–pest interactions is not yet comprehensive. Future research endeavors hold the potential to expand our knowledge by uncovering additional UPS targets and elucidating the mechanisms by which they influence plant defense responses against pests.

#### 2.2.2. Autophagy

Autophagy is a conserved eukaryotic process in which cytoplasmic materials and damaged organelles are recycled or degraded inside a lytic cellular compartment to maintain homeostasis [66,67]. Compared to UPS, which degrades individual proteins, autophagy can eliminate specific proteins, macromolecular complexes, or even whole organelles and pathogens [68]. Several autophagy-related genes (ATGs) and proteins in plants play crucial roles in response to pest attacks by modulating defense signaling, nutrient recycling, and cell death. Autophagy is executed through coordinated action of autophagy-related (ATG) proteins. Selective autophagy is regulated through specific interactions of autophagy cargo receptors and ATG8 proteins [66]. In plants, more than 40 ATG genes have been identified, which have distinct yet collaborative roles in mediating autophagy [9]. NBR1 is a selective autophagy cargo receptor and interacts with ATG8 proteins to facilitate the autophagy-dependent degradation of target proteins [8,69].

Recent studies have demonstrated that autophagy-related genes and proteins play crucial roles in plant responses to pest attacks. For instance, infestation by the green peach aphid (*Myzus persicae*) induces the expression of autophagy-related (ATG) genes and increases the formation of autophagosomes. Aphid reproduction was significantly reduced on two autophagy-deficient mutants, *atg5.1* and *atg7.2*, compared to the wild type, suggesting that autophagy facilitates aphid infestation [70]. The expression of *OsAtg4* and *OsAtg7* is also observed to increase significantly in the roots of resistant rice plants upon *Meloidogyne graminicola* infection [71]. Autophagy is also intricately linked with phytohormone signaling, playing crucial roles in modulating plant development and stress responses [72]. Tomato selective autophagy receptor NBR1a positively regulates the resistance to RKNs. NBR1a mediates the accumulation of JA and JA-Ile content and the increase of antioxidant enzyme activity in *Solanum lycopersicum* against RKNs [73]. Autophagy is also reported to promote JA-mediated defense against RKNs by forming a positive feedback circuit in the degradation of JA negative regulator JASMONATE-ASSOCIATED MYC2-LIKE (JAMs) and transcriptional activation by ERF1 [59]. In a recent study, an effector from the phloem-feeding insect brown planthopper (*Nilaparvata lugens*), BPH14-interacting salivary protein (BISP), was found to be recognized and bound by the nucleotide-binding leucine-rich repeat receptor BPH14 on resistant rice plants. BPH14 enhanced the interaction of OsNBR1 with BISP and controls BISP levels by OsNBR1-mediated autophagic degradation to fine-tune rice resistance [74]. Whether the levels of other insect effector proteins are also regulated by autophagy remains to be investigated.

The UPS is involved in rapid destruction of abnormal peptides and short-lived cellular regulators, which, in turn, control many processes [75,76]. It is highly selective and fast-acting, making it crucial for early responses to pest attacks. It primarily targets short-lived regulatory proteins, such as transcription factors and signaling components, for degradation. This allows plants to fine-tune hormone signaling pathways like JA and SA, which are pivotal in pest defense. For example, the degradation of repressors in the hormone pathway is critical for activating pest-responsive genes. While autophagy is a complicated self-eating process, which is probably more important during extended stress periods, including nutrient deprivation, drought, and pathogen attack. It facilitates the recycling of damaged organelles and large protein complexes, helping maintain cellular homeostasis [77,78]. The knowledge of accurate regulatory networks of autophagy during plant–pest interaction is still limited. The UPS and autophagy are not mutually exclusive but complementary. A deeper understanding of how these pathways interact in the context of pest resistance could reveal strategies to enhance plant immunity by modulating these systems.

## 3. Plant Proteins Targeted by Pests

Pests have evolved to secrete effectors to manipulate and subvert host cell protein to their advantage [12]. By hijacking the essential cellular pathways, they create a conducive environment for their invasion and evade host immune responses. In contrast to pathogenic bacteria, fewer instances of plant proteins targeted by pests have been documented. In this review, we mainly focus on the primary components that insect and nematode effectors target to alter the host’s proteome (Figure 2). Readers are referred to several reviews for more detailed advances in insect and nematode effectors [79,80,81]. These components mainly belonged to the pathways discussed above: the UPS components in the UPS system; the autophagy-related proteins in the autophagy system; the phytohormone signaling components; and transcriptional factors in the downstream signaling pathways. These components are important factors that plants rely on to shape their proteome (Figure 2; Table 1). Through this mechanism, pests efficiently manipulate the composition of plant proteins and finally the plant immune responses.

### 3.1. UPS Components

The UPS serves as a major regulator of plant protein homeostasis and plays a crucial role in controlling hormone pathway switches. A significant number of pathogen effectors have been identified to target the UPS components, aiming to enhance their invasion capabilities [49,50,95,96]. Recent studies indicate that the UPS components are also targets of pest effectors, especially the E3 ligases. Liu et al. found that an effector Mp64 from the major aphid pest, *Myzus persicae*, and the effector CRN83_152 from oomycete pathogen, *Phytophthora capsici*, both target the host immune regulator SIZ1, an E3 SUMO ligase. Mp64 increases SIZ1 protein levels in transient assays, while *P. capsici* effector CRN83_152 enhances SIZ1-E3 SUMO ligase activity in vivo. These interactions promote host susceptibility, with *Arabidopsis* mutants lacking SIZ1 showing reduced susceptibility to both pests. The findings highlight a shared virulence strategy, where distinct pests target central host proteins to suppress immunity and promote infection [82]. GpRbp-1 is another effector from the potato cyst nematode *Globodera pallida* that interacts with a functional potato homologue of the Homology to E6-AP C-Terminus (HECT)-type ubiquitin E3 ligase UPL3 [83]. The outcome of this interaction is yet to be fully characterized; however, there are reports indicating that HECT-containing ubiquitin-protein ligases (UPLs) serve as regulators of the proteasome and play a role in plant immunity [83]. GpRbp-1 may inhibit or activate UPL3 to regulate the transcription of defense-related genes to promote susceptibility, as a number of genes differentially expressed in association with *upl3* are linked to stress responses and metabolism. Numerous genes that code for components of the UPS exhibited varying regulation in syncytia induced by *Heterodera glycines* in both susceptible and resistant soybean plants [97]. This also implies the participation of the host UPS in both syncytium formation and defense against nematode infection. Cyclophilin effector Al106 of the mirid bug *Apolygus lucorum* was recently found to interact with the U-box E3 ubiquitin ligase PUB33 in *N. benthamiana* and *Arabidopsis thaliana* and inhibit its ubiquitination [84]. A secreted effector from the potato cyst nematode *Globodera rostochiensis* named GrUBCEP12, when lacking the signal peptide (Gr^ΔSP^UBCEP12), is processed into free ubiquitin and a CEP12 peptide (GrCEP12) *in planta*. RPN2a, a gene encoding the subunit of the 26S proteasome, is dramatically suppressed in Gr^ΔSP^UBCEP12 over-expression plants [98].

Like pathogens, pests also evolved E3 ligase effectors that usurp host ubiquitin-related cellular processes and interfere with defense responses for their own benefit. The SSGP-71 effector gene family is the largest known arthropod gene family found within the wheat pest *Mayetiola destructor.* SSGP-71 genes encode E3-ubiquitin-ligase mimics; it is very likely that the Hessian fly SSGP-71 effectors will function as plant F-box mimics and influence the turnover of plant proteins critical for host plant immunity via the 26S proteasome. Supporting this proposition, the Hessian fly SSGP-71-142 has demonstrated interaction with wheat SKP6, a component of the SKP-Cullin-F-box E3 ubiquitin ligase complex responsible for targeting proteins for degradation, as revealed by a yeast two-hybrid [85].

### 3.2. Autophagy-Related Proteins

As an important part of the degradation system, autophagy stands as a pivotal regulator in plant innate immunity [8,67]. Concurrently, it is noteworthy that several pathogens and pests have not remained passive players in this biological interplay. They have evolved sophisticated measures to specifically target and exploit the autophagic systems of plants to their advantage during infection [8,67,86]. In the above-mentioned example, BISP plays a crucial role in the feeding and performance of BPHs and suppresses plant defense on susceptible rice plants [74]. BISP interacts with OsNBR1, and its level is controlled by OsNBR1-mediated autophagic degradation in BPH14-contaoning resistant plants [74]. It is intriguing to note that the level of BISP protein accumulates during feeding on susceptible rice plants. Whether this accumulation is accompanied by the effector inhibition of autophagy, akin to strategies employed by phytopathogen effectors, remains to be investigated. In a yeast two-hybrid (Y2H) screen between 25 *Arabidopsis* ATG proteins and 184 effectors from bacterial, fungal, oomycete, and nematode, researchers also identified several effectors (Rbp001/2/3/5) from the cyst nematode *Globodera pallida* that interact with different ATGs, including ATG1, ATG8, ATG12, and ATG20 [86]. A similar situation was observed in the cyst nematode effector Nematode Manipulator of Autophagy System 1 (NMAS1); it also interacts with host plant ATG8 proteins [87]. The outcome of these interactions was not characterized. It may either be targeted by autophagy-mediated degradation to dampen the infection, or they can enhance or inhibit key steps in autophagy to benefit the infection. More studies are needed to elucidate the function of autophagy in plant–pest interactions.

### 3.3. Phytohormone Signaling Components

Plant hormone signaling plays a pivotal role in coordinating plant resistance against pests [55,56]. Therefore, maintaining homeostasis of key hormone components is crucial for the normal functioning of hormone signaling. Given that protein homeostasis is essential for the activation of plant hormone signaling, numerous pathogens adeptly exploit this vulnerability by targeting components of hormone signaling, thus hindering the host’s defensive responses [99,100]. Pests are reported to similarly capitalize on this strategy. HARP1 is an effector from cotton bollworm (*Helicoverpa armigera)* oral secretion and was released to cotton leaves during feeding. HARP1 directly interacted with multiple JASMONATE-ZIM-domain (JAZ) repressors, hindering the COI1-mediated degradation of JAZ and thereby impeding the transduction of JA signaling [58]. Some effectors have unidentified target proteins within plants, but they are known to modulate defense-related hormones such as SA, JA, or abscisic acid. For example, Bt56, a whitefly-secreted low molecular weight salivary protein, when overexpressed in plants, results in eliciting the SA-signaling pathway, thus promoting susceptibility [90]. Two effectors from the two-spotted spider mite (*Tetranychus urticae*) named tetranin1 (Tet1) and tetranin2 (Tet2) also induced JA, SA, and abscisic acid biosynthesis in kidney bean [101]. The root-knot nematode effector MiMSP32 targets host 12-oxophytodienoate reductase 2 (OPR2), a key player in the jasmonic acid (JA) biosynthesis pathway to regulate plant susceptibility [88]. Overall, these cases underscore the intricate mechanisms pests employ to manipulate plant hormone signaling, revealing a crucial nexus where effector-target interactions disrupt hormonal balance to compromise plant defense systems.

### 3.4. Transcriptional Factors

Transcription factors (TFs) serve as a crucial determinant in shaping the dynamics of gene transcription. In most cases, the transcript level also determines the protein level. Optimal protein levels are essential for ensuring the formation of transcription factor-DNA complexes, enabling them to bind to specific genomic regions with precision. Thus, TFs are favorable targets of pathogens and pest effectors to modulate plant homeostasis. The whitefly *Bemisia. tabaci* effector Bsp9 accumulates in the cytoplasm, where it interacts with the *Arabidopsis* transcription factor WRKY33, hindering its nuclear localization. This way represents another strategy to control protein levels, consequently impeding the downstream immune signaling pathways [89]. Bt56, an ortholog of Bsp9, also interacts with a transcriptional factor, the KNOTTED 1-like homeobox (KNOX) transcription factor NTH202, in punctate structures in tobacco cytoplasm. This localization implies that, similar to Bsp9, Bt56 hinders the movement of a transcription factor to the nucleus, thereby impeding its function [90]. A C-terminal polypeptide of vitellogenin (VgC) in the small brown planthopper (*Laodelphax striatellus*), when secreted into rice plants, was found to interact directly with the rice transcription factor OsWRKY71 and repress its transcription regulatory activity to attenuate host rice defenses [91]. The *Heterodera schachtii* effector Hs10A07 was shown to interact with IAA16, an Aux/IAA transcription factor, to modulate ARF expression [92].

### 3.5. Other Signaling Components

In addition to the above-mentioned primary components, other proteins are also targeted by pest effectors, including enzymes involved in metabolic processes, regulatory proteins that modulate signaling pathways, and structural proteins that maintain cellular integrity. Although these effectors do not fall into a single functional category, they share a common ability to modulate host protein homeostasis, demonstrating the diverse strategies pests use to manipulate plant biology for their benefit. These additional targets further broaden the range of cellular functions that pests can manipulate to suppress plant immunity and enhance their own survival. For example, the effector Mp1 from *Myzus persicae* was found to associate with *Arabidopsis* and potato Vacuolar-Protein-Sorting-Associated Protein 52 (VPS52), a component of the Golgi-Associated-Retrograde Protein (GARP) complex. AtVPS52 and StVPS52 protein levels are reduced upon aphid infestation. Although no evidence of a role for Mp1 in VPS52 degradation was shown, a negative relevance of VPS52 protein level and aphid virulence was observed, indicating aphid-mediated degradation of this protein may be an important step during infestation [93]. The *Bemisia tabaci* effector BtFTSP1 can associate with the defensive ferredoxin 1 (NtFD1) in *Nicotiana tabacum* and destabilize NtFD1 by disassociating the NtFD1 polymer in plant cytosol, leading to the degradation of NtFD1 in a ubiquitin-dependent manner [94].

## 4. Regulation of Plant Host Proteins by Insect-Borne Microbes

A diverse array of insects plays a pivotal role as carriers, or vectors, for numerous viral and pathogenic microbes, thereby serving as conduits for the efficient transmission of these microbes. Effectors from these viral and pathogenic microbes were also documented to perturb plant protein homeostasis, conferring advantages to their insect vectors residing on host plants [102,103]. Phytoplasmas are insect-transmitted phytopathogenic bacteria that can induce changes in plant morphology as well as influence the longevity, reproduction rates, and behavior of their insect vectors. The phytoplasm proteins SAP11 and SAP54 target plant proteins for degradation, resulting in promotion of insect vector colonization. SAP11 binds and destabilizes *Arabidopsis* TCP transcription factors to down-regulate LOX2 expression and JA synthesis [104]. While SAP54 mediates degradation of the MADS-domain transcription factor (MTF) family by interacting with the RADIATION SENSITIVE23 (RAD23) family proteins [105]. The rice orange leaf phytoplasma effector SRP1 can bind to glutamine synthetase, disrupting its decamer formation and enzymatic activity, which impairs chlorophyll precursor biosynthesis and causes rice leaf yellowing, potentially enhancing leafhopper vector attraction and pathogen transmission [106]. The tomato yellow leaf curl virus (TYLCV) protein C2 interacts with and inhibits the ubiquitin precursor protein RPS27A, preventing the ubiquitination and degradation of JAZ1. This leads to the suppression of MYC2 and JA-responsive terpene synthase genes in plants expressing the C2 protein from TYLCV, ultimately enhancing whitefly performance [107]. The same JAZ degradation repression was reported by the *Myzus persicae*-borne cucumber mosaic virus (CMV) 2b protein through direct interaction [108].

## 5. Concluding Remarks and Future Directions

The regulation of protein levels within the plant plays a pivotal role in modulating the plant’s immune response to pests. Conversely, pests and their associated microbes secrete a diverse array of effector proteins to subvert plants’ immune responses for their invasion and colonization. Despite significant advancements in understanding protein regulation in plant–pest interactions, many more knowledge gaps remain. On one hand, more plant immune components and molecular mechanisms need to be deciphered during plant–pest interactions. Identifying key regulatory nodes and the factors that influence their activation or suppression is essential for developing targeted interventions. On the other hand, there is a need for in-depth exploration into the diverse repertoire of effector proteins employed by pests and their targets in plants. Advanced tools like AlphaFold-Multimer and other computational prediction models hold great potential for identifying effector proteins and their specific plant targets [109,110]. Understanding the structural and functional aspects of these effectors, especially their specific interactions with plant proteins, can uncover vulnerabilities in the pest’s strategies and potentially inform the development of novel pest control measures. Furthermore, there is a need to explore the combination of pest-inducible regulatory elements to engineer plants with a stringent expression of defense proteins. This approach is essential to ensuring that plants can effectively activate their defense mechanisms upon pest invasion without compromising their overall fitness. Investigating the dynamics of protein regulation can contribute to a more nuanced understanding of plant defense strategies.

## Figures and Tables

**Table 1:** Representative direct protein-protein interaction partners from pests and plants listed in this paper.

	Effector	Pest Species	Plant Protein	Plant Species	Interaction Outcome	Reference
UPS components	Mp64	*Myzus persicae*	SIZ1	*Arabidopsis*	increase SIZ1 protein levels	[82]
	CRN83_152	*Phytophthora capsici*	SIZ1	*Nicotiana benthamiana*	enhance SIZ1-E3 SUMO ligase activity	[82]
	GpRbp	*Globodera pallida*	UPL3	*Solanum tuberosum*	unknown	[83]
	Al106	*Apolygus lucorum*	PUB33	*Nicotiana benthamiana*	inhibit PUB33 ubiquitination	[84]
	SSGP-71	*Mayetiola destructor*	SKP6	wheat	unknown	[85]
Autophagy-related proteins	BISP	*Nilaparvata lugens*	NBR1	*Oryza sativa*	degradation of BISP	[74]
	Rbp001/2/3/5	*Globodera pallida*	ATG1/8/12/20	*Arabidopsis*	unknown	[86]
	NMAS1	*Heterodera* and *Globodera* spp.	ATG8	*Solanum tuberosum*	supress ROS	[87]
Phytohormone signaling components	HARP1	*Helicoverpa armigera*	JAZ proteins	*Arabidopsis*, Cotton, and Tobacco	stabilize JAZs	[58]
	MiMSP32	*Meloidogyne incognita*	OPR2	*Solanum lycopersicum*	increase in the concentration of 12-OPDA	[88]
Transcriptional factors	Bsp9	*Bemisia tabaci*	WRKY33	*Arabidopsis*	decrease WRKY33 in nuclear	[89]
	Bt56	*Bemisia tabaci*	NTH202	*Nicotiana benthamiana*	decrease NTH202 in nuclear	[90]
	VgC	*Laodelphax striatellus*	OsWRKY71	*Oryza sativa*	repress OsWRKY71 activity	[91]
	Hs10A07	*Heterodera schachtii*	IAA16	*Arabidopsis*	modulate ARF expression	[92]
Other signaling components	Mp1	*Myzus persicae*	VPS52	*Arabidopsis*	decrease VPS52 protein level	[93]
	BtFTSP1	*Bemisia tabaci*	NtFD1	*Nicotiana tabacum*	destabilize NtFD1	[94]

## Data Availability

All data have been included in the manuscript.
